# Biomarkers in Colorectal Cancer: The Role of Translational Proteomics Research

**DOI:** 10.3389/fonc.2019.01284

**Published:** 2019-11-27

**Authors:** Bruno Augusto Alves Martins, Gabriel Fonseca de Bulhões, Igor Norat Cavalcanti, Mickaella Michelson Martins, Paulo Gonçalves de Oliveira, Aline Maria Araújo Martins

**Affiliations:** ^1^Medical Sciences Postgraduate Program, School of Medicine, University of Brasilia, Brasília, Brazil; ^2^UniCeub—Centro Universitário Do Distrito Federal, Translational Medicine Group, School of Medicine, Brasilia, Brazil; ^3^Department of Cell Biology, Institute of Biology, University of Brasilia, Brasilia, Brazil; ^4^Metabolomics and Bioanalysis Center, San Pablo CEU University, Madrid, Spain

**Keywords:** colorectal cancer, biomarkers, translational research, proteomics, mass spectrometry

## Abstract

Colorectal cancer is one of the most common cancers in the world, and it is one of the leading causes of cancer-related death. Despite recent progress in the development of screening programs and in the management of patients with colorectal cancer, there are still many gaps to fill, ranging from the prevention and early diagnosis to the determination of prognosis factors and treatment of metastatic disease, to establish a personalized approach. The genetic profile approach has been increasingly used in the decision-making process, especially in the choice of targeted therapies and in the prediction of drug response, but there are still few validated biomarkers of colorectal cancer for clinical practice. The discovery of non-invasive, sensitive, and specific biomarkers is an urgent need, and translational proteomics play a key role in this process, as they enable better comprehension of colorectal carcinogenesis, identification of potential markers, and subsequent validation. This review provides an overview of recent advances in the search for colorectal cancer biomarkers through proteomics studies according to biomarker function and clinical application.

## Introduction

Colorectal cancer (CRC) is the third most commonly diagnosed cancer among adults and is the third leading cause of cancer-related death in the United States (1). Most colorectal cancers occur sporadically and are characterized by a sequenced carcinogenesis process that involves the progressive accumulation of mutations in a period that lasts on average 10–15 years (2–5). This long evolution interval allows for the successful application of screening, early detection of cancer, and removal of premalignant lesions (adenomas), leading to a reduction in incidence and mortality (5–8). Despite the opportunity for early diagnosis, ~20–25% of CRC cases are diagnosed at stage IV, when the patients have already presented with distant metastasis and the 5-year survival rate is <10%. In contrast, the 5-year survival for patients with early localized disease, when surgical resection is possible, may be as high as 90% (9, 10).

The current gold standard screening strategy is through a colonoscopy. The guidelines recommend that individuals aged 45 years and older with an average risk of CRC undergo regular screening (8). However, colonoscopies have poor patient compliance. The procedure is expensive and invasive and carries risks, such as hemorrhage, colonic perforation, and cardiorespiratory complications. Other reasons for low adherence are related to a preoccupation with pudency, procedure discomfort, and bowel preparation (11). The most frequently used non-invasive screening method is the guaiac fecal occult blood test (gFOBT), based on the identification of hemoglobin peroxidase activity in the stool. Although FOBT is an easy and cost-effective method for screening CRC, it has relatively poor selectivity and sensitivity, resulting in high rates of both false positives and false negatives (4, 5).

**Figure 1:**
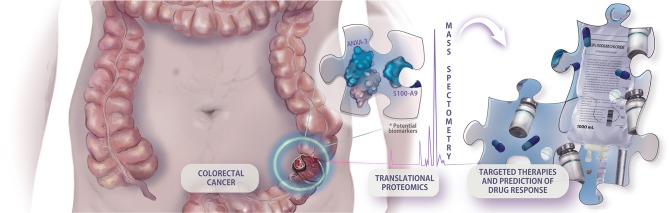
Example of hypothetical application of translational proteomic research in colorectal cancer approach. The prospection of new predictive biomarkers is cardinal to the implementation of an integrative and personalized medicine, making possible the individual assessment of targeted therapies, and drug response.

Therefore, alternative cost-effective, non-invasive, easily measurable, and accurate screening procedures are urgently required for CRC screening. Thus, the clinical applications of biomarkers in CRC are not only needed for the early detection of the disease but are also essential for prognostic stratification, surveillance, and therapy selection (Figure 1) (12–14). The increasing emergence of adjuvant and neoadjuvant therapy approaches results in an urgent need for predictive biomarkers that guide the decision-making process (12). An example of the importance of predictive biomarkers is how treatment with drugs can antagonize the epidermal growth factor receptor (EGFR) in patients with KRAS-wild-type tumors. The discovery of this targeting therapy made the determination of KRAS status a mandatory step for the adequacy of chemotherapy in patients with advanced colorectal cancer (15).

Recent progress in genomics, transcriptomics, proteomics, and metabolomics has expanded the number of candidate biomarkers and led to better comprehension of the progression of colorectal cancer as well as the identification of molecular signatures (16–22).

Dysplastic and neoplastic tissues regulate the expression of proteins and generate protein profiles that may be associated with the progression of these lesions in many different and interacting signaling pathways (23). Proteomics represents a large number of approaches employed for large-scale recognition, measurement, characterization, and analysis of proteins. The majority of studies on biomarker discovery employ quantitative mass spectrometry-based techniques for the identification and validation of dysregulated proteins as disease biomarker candidates (24). Translational proteomics research emphasizes the translation of general proteomics science to determine protein expression profiles that generate pathogenic phenotype variations and contribute to clinical practice (15).

This review aims to provide an overview of recent advances in mass spectrometry-based proteomics in the search for protein biomarkers of CRC with the potential for clinical application according to biomarker functions: diagnostic, predictive, or prognostic.

## Diagnostic Biomarkers

A diagnostic biomarker can be defined as a biological characteristic that detects or suggests the presence of a disease or condition of interest or identifies an individual with a subtype of the disease (25).

It is well-established that colorectal cancer screening strategies that lead to the identification and removal of adenomatous polyps and other premalignant lesions result in a decrease in CRC mortality (26). Colonoscopies are the only screening method that can identify and remove precancerous polyps; however, the exam requires bowel preparation and dietary modification, it is operator dependent, and it has been associated with major complications, such as cardiopulmonary events, gastrointestinal bleeding, and perforation (27). Perforation is the most frequent major complication, occurring in 0.016–0.8% of diagnostic examinations and up to 5% of therapeutic colonoscopies (28).

Some studies report the non-attendance rate of colonoscopies to be 10–20% after a positive fecal occult blood test (29, 30). The main factors associated with non-adherence with colonoscopies are laxative bowel preparation, lack of awareness of the significance of screening, and concerns about embarrassment, modesty, and dignity (31). Plumb et al. (32) evaluated the explanations for colonoscopy non-participation, and ~30% of the patients addressed the unwillingness to undergo the test as the major barrier to go through with the whole screening program.

Non-invasive methods such as fecal immunochemical tests, gFOBT, and stool DNA tests can be used for regular screening, but positive results should be followed up with timely colonoscopy (8). The current fecal occult blood test methods are more easily accepted by participants in population screening programs; however, they are subject to various interfering factors with some causes of false-negative, false-positive results, and low sensitivity rates for detecting colon polyps (33–35) Therefore, early, non-invasive, specific, and sensitive biomarkers are still required for screening strategies in colorectal cancer.

Many proteomic approaches have been used in the search for potential diagnostic biomarkers. Ghazanfar et al. (36) performed two-dimensional gel electrophoresis coupled with mass spectrometry for the expression profiling of proteins extracted from freshly frozen human colorectal cancer tissue specimens (12 patients) and neighboring non-tumor tissue, and they demonstrated the upregulation of some proteins, such as actin beta-like 2 (ACTBL2), in colorectal cancer. Hao et al. (37) used high-resolution Fourier transform mass spectrometry to evaluate 22 pairs of cancerous and adjacent normal tissue specimens that were gathered from 22 individuals and revealed an overexpression of dipeptidase 1 (DPEP1) in colorectal tumor tissue.

Formalin-fixed paraffin-embedded (FFPE) tissues can also be used in proteomics approaches, allowing access to archival samples, allowing usage of larger cohorts and more robust analyses, and optimizing the follow-up data of patients' clinical conditions. Quesada-Calvo et al. (23) analyzed 76 formalin-fixed paraffin-embedded colorectal tissues from early CRC stages (pT1N0M0 and pT2N0M0), as well as normal or inflamed mucosa, by label-free proteomics, and different expression levels of olfactomedin-4 (OLFM4), kininogen-1 (KNG1), and transport protein Sec24C (Sec24C) were observed in the early CRC stages compared to normal and premalignant tissues. Although the experiment was performed with liquid chromatography-mass tandem mass spectrometry (LC-MS/MS), the results were also validated by immunohistochemistry of these annotated effectors. Yamamoto et al. (38) also used formalin-fixed and paraffin-embedded (FFPE) CRC tissue to perform liquid chromatography (LC)/mass spectrometry (MS) based on a global proteomic approach, revealing higher expression levels of cyclophilin A, annexin A2, and aldolase A in cancer compared to non-cancer regions (38).

Blood-based biomarkers are potentially the best matrices for early diagnosis and surveillance of colorectal cancer because the specimens can be obtained easily by a non-invasive method with minimal cost and risk (24, 39). Ivancic et al. (40) used targeted liquid chromatography-tandem mass spectrometry to analyze blood from 213 healthy individuals and 50 patients with non-metastatic CRC. This approach resulted in a panel of five proteins (leucine-rich alpha-2-glycoprotein 1, EGFR, inter-alpha-trypsin inhibitor heavy-chain family member 4, hemopexin, and superoxide dismutase 3) with good performance for CRC detection, which present 89% specificity at over 70% sensitivity in the validation set. Bhardwaj et al. (41) also proposed a protein panel for the early detection of CRC, utilizing an approach with liquid chromatography/multiple reaction monitoring-mass spectrometry and a subsequent proximity extension assay to analyze plasma from 96 CRC patients and 94 controls. They demonstrated promising CRC-screening performance of a five-marker blood-based profile consisting of mannan binding lectin serine protease 1, osteopontin, serum paraoxonase lactonase 3, transferrin receptor protein 1, and amphiregulin.

Yu et al. (42) used magnetic beads and matrix-assisted laser-desorption/ionization time-of-flight (MALDI-TOF) mass spectrometry to analyze 127 CRC serum samples and 90 healthy control serum samples. The protein serine/threonine kinase 4 (STK4 or MST1) was identified by tandem mass spectrometry (MS/MS) and validated with Western blotting and an enzyme-linked immunosorbent assay (ELISA). They demonstrated a downregulation of MST1 in CRC patients, with a sensitivity of 92.3% and specificity of 100% in the diagnosis of colorectal cancer when gathered with carcinoembryonic antigen and FOBT. Their work also implied that MST1 could be a predictive marker for distant metastasis (42).

Fan et al. (43) also conducted a study with serum samples that were analyzed by a combination of high-performance liquid chromatography and mass spectrometry and further validation with Western blotting. They verified an upregulation of macrophage mannose receptor 1 (MRC1) and S100 calcium-binding protein A9 (S100A9) in colorectal cancer. Members of the serpin family, such as SERPINA1 (alpha-1-antitrypsin, A1AT), SERPINA3 (alpha-1-antichymotrypsin, AACT), and SERPINC1 (antithrombin-3, AT-III), have also been described as potential biomarkers of adenomatous polyps and colorectal carcinomas through analyses of serum samples by multiplexed quantification with an isobaric tag for relative and absolute quantitation (iTRAQ) (44).

Despite the expansion of MS-based proteomics research and the large number of diagnostic biomarker candidates (Table 1 shows some examples of candidates for diagnostic protein biomarkers), none of them were successfully translated into clinical practice. This probably occurs due to the difficulty of validating the possible biomarkers in large cohorts and comparing the results with the current screening methods. However, the continuation of proteomic research is essential because, certainly, there is a space in the CRC screening that needs to be filled by reliable biomarkers.

**Table 1:** Examples of candidate diagnostic biomarkers.

**References**	**Biomarker/Regulation**	**Technique**	**Sample**
Ghazanfar et al. (36)	-Actin beta-like 2 (ACTBL2)	Two-dimensional gel electrophoresis coupled to mass spectrometry (2DE-MS)	CRC tissue
Hao et al. (37)	-Dipeptidase 1 (DPEP1)	Fourier transform mass spectrometry (FTMS)	CRC tissue
Quesada-Calvo et al. (23)	- Olfamectomedin-4 (OLFM4) - Kininogen-1 (KNG1) - Transport protein Sec-24 (Sec-24)	Liquid chromatography–mass spectrometry (LC-MS). Further immunohistochemistry validation.	FFPE CRC tissue
Yamamoto et al. (38)	- Cyclophilin A - Annexin A2 - Aldolase A	LC-MS	FFPE CRC tissue
Ivancic et al. (40)	- Leucine-rich alpha-2-glycoprotein 1Epidermal growth - factor receptor - Inter-alpha-trypsin inhibitor heavy-chain family member 4 - Hemopexin - Superoxide dismutase 3	Targeted liquid chromatography-tandem mass spectrometry	Serum
Bhardwaj et al. (41)	- Mannan binding lectin serine protease 1 - Osteopontin - Serum paraoxonase lactonase 3 - Transferrin receptor protein 1 - Amphiregulin	Liquid chromatography/multiple reaction monitoring-mass spectrometry and proximity extension assay	Plasma
Yu et al. (42)	- Serine/threonine kinase 4 (STK4 or MST1)	Mass spectrometry (MS/MS). Also verified with Western blotting and enzyme-linked immunosorbent assay (ELISA).	Serum
Fan et al. (43)	- Macrophage mannose receptor 1 (MRC1) - S100 calcium-binding protein A9 (S100A9)	High-performance liquid chromatography (HPLC) and Western blotting.	Serum
Peltier et al. (44)	- Alpha-1-antitrypsin (SERPINA 1) - Alpha-1 antichymotrypsin (SERPINA 3) - Antithrombin-3 (SERPINC1)	Multiplexed quantification with isobaric tag for relative and absolute quantitation (iTRAQ)	Serum

## Predictive Biomarkers

The predictive biomarkers are used to indicate the response to a specific treatment and to guide the decision-making process. The prospection of new predictive biomarkers is crucial to the evolution of the management of patients with colorectal cancer in the near future, and proteomics represent a powerful strategy for the discovery and implementation of personalized approaches. The increasing number of chemotherapy and immunotherapy drugs and the emergence of target therapies make it necessary to discover some response parameters and monitoring evaluations (45, 46).

Concerning the individualized and integrative treatment of patients with colorectal cancer, the understanding of the mechanism underlying chemotherapy resistance is a prerequisite to overcome the resistance and improve the efficacy of chemotherapy. In addition, the identification of good-responder patients is also important to guide and improve personalized therapies. Wang et al. (47) correlated the capacity of proteomic, genomic, and transcriptomic profiles to predict drug sensitivity. Forty-four CRC cell lines were analyzed by liquid chromatography-tandem mass spectrometry (LC-MS/MS)-based shotgun proteomics and compared against 90 colorectal cancer primary tumor specimens and 60 normal tissue biopsies. The proteomic profile was compared on mutations, DNA copy number, and mRNA expression, and the results showed that proteomic data tended to exhibit better potential for predicting sensitivity to 5-fluorouracil, SN-38, erlotinib, regorafenib, and oxaliplatin when compared to genomic and transcriptomic profiles (47).

Guo et al. (48) investigated protein elements that might be implicated in oxaliplatin resistance by comparing the proteome between oxaliplatin-sensitive HT-29 wild-type cells and oxaliplatin-resistant HT-29 cells using 2D gel electrophoresis followed by MALDI TOF/TOF mass spectrometry. It was observed that poly(C)-binding protein 1 (PCBP1) expression was significantly more elevated in tumor samples from oxaliplatin-refractory patients than in those from responsive patients, suggesting that PCBP1 is a protein marker of oxaliplatin resistance in colorectal cancer cell cultures.

Martin et al. (49) evaluated the response to vascular endothelial growth factor inhibitor (bevacizumab) in patients with metastatic colorectal cancer through the analysis of pretreatment serum from 23 patients. 2D difference gel electrophoresis (2D-DIGE) was performed, followed by LC-MS/MS, which identified 68 differentially expressed proteins between responders and non-responders. Three proteins, apolipoprotein E (APOE), angiotensinogen (AGT), and vitamin D-binding protein (DBP), were chosen for validation through immunohistochemistry and enzyme-linked immunosorbent assay (ELISA) and were correlated with better survival outcomes in patients treated with chemotherapy and bevacizumab (49).

The response to EGFR-targeted therapies was also evaluated by Katsila et al. (50) through a quantitative proteomic analysis of the plasma of patients with metastatic colorectal cancer compared with the 3D colorectal cancer spheroid secretome (isogenic cells SW48) of patients treated with cetuximab. They showed that the plasma level of phosphorylated-EGFR (pEGFR) was associated with sensitivity to cetuximab therapy, suggesting that pEGFR could be a predictive drug-response biomarker (50).

An expanding research area due to tailored-made therapy for patients with colorectal cancer is the therapeutic targets in anti-tumor immunity (51). Studies with immune checkpoint-inhibiting drugs, such as those directed against cytotoxic T-lymphocyte antigen 4 (CTLA-4) and programmed death-1 receptor (PD1) and its ligand PD-L1, have demonstrated promising results in the therapy of patients with metastatic colorectal cancer (52–54).

Until now, the best indicator of responsiveness to immunotherapy in patients with colorectal cancer seems to be mismatch repair deficiency (55). Repair system deficiency leads to a high burden of somatic mutations, which increases the immunogenicity (51).

Furthermore, tumors with high microsatellite instability (MSI-H) present a dense Th1 lymphocytic infiltration and a cytokine-rich microenvironment that is related to the highly upregulated expression of multiple immune checkpoint proteins (56). Unfortunately, the patients with MSI-H tumors represent only a subgroup of the patients with colorectal cancer, and the likelihood of mismatch repair deficiency varies according to the stage of the disease, reaching 4–5% in the metastatic disease. In addition, not all patients with MSI-H tumors respond to immunotherapy (57).

Therefore, a complete understanding of the response of the immune system to MSI-H is crucial to optimizing the immunotherapy approach. Some studies have demonstrated promising prognostic biomarkers, such as the expression of heat shock protein 110 and protein ß2-microglobulin, to stratify patients with MSI-H CRC according to prognosis (58, 59).

In this scenario, the application of mass spectrometry-based immune-proteomic methods is a powerful tool in the search for overexpressed immunogenic proteins that could be new targets of immunotherapeutic development. Yang et al. (60) used mass spectrometry to evaluate antibody-reactive proteins, and this was followed by Western blotting and immunohistochemistry validation. Their experiment described differential expression of proteasome subunit alpha type 1 (PSA1), leucine aminopeptidase 3 (LAP3), annexin A3 (ANXA3), and maspin (serpin B5), demonstrating a proteomic profile of antibody-inducing cancer-associated immunogens (60). In another study with an immuno-proteomic approach by the same group, overexpression of olfactomedin 4, CD11b, and integrin alpha-2 was identified in the tumor tissue of patients with colorectal cancer with liver metastases (61).

The current treatment of locally advanced rectal cancer (stages II and III) is neoadjuvant chemoradiation followed by surgery (62). The main role of neoadjuvant therapy is local tumor control, but, in ~10–20% of patients, a pathologic complete response is observed. This fact allows for the possibility of a selective surgical approach, which was described in Habr-Gama et al. (63). One of the most challenging issues in the modern management of patients with rectal cancer is to predict the response to neoadjuvant therapy. Recently, Chauvin et al. (64) highlighted different protein signatures in patients who underwent neoadjuvant therapy in a study on mass spectrometry of formalin-fixed paraffin-embedded tumor biopsies. The researchers identified that interferon-induced protein with tetratricopeptide repeats 1 (IFIT1), FAST kinase domains 2 (FASTKD2), phosphatidylinositol-5-phosphate 4-kinase type-2 beta (PIP4K2B), AT-rich interactive domain-containing protein 1B (ARID1B), and solute carrier family 25 member 33 (SLC25A33) were overexpressed in the tumor tissue of the initial biopsy from patients who achieved complete response to neoadjuvant chemoradiotherapy. In the non-responder group, they identified that caldesmon 1 (CALD1), carboxypeptidase A3 (CPA3), beta-1,3-galactosyltransferase 5 (B3GALT5), CD177, and receptor-interacting serine/threonine-protein kinase 1 (RIPK1) were overexpressed (64).

The predictive protein biomarkers face the same problem of slow translation to clinical application as the diagnostic biomarkers. Table 2 shows some examples of candidates for predictive protein biomarkers. The pursuit for new biomarkers maintains a central role in the development of the integrative management of CRC patients because it is crucial to determine the responses to neoadjuvant and adjuvant therapies. Mass spectrometry's ability to detect low-abundance elements makes this technique a powerful tool for prospecting these potential biomarkers.

**Table 2:** Examples of candidate predictive biomarkers.

**References**	**Biomarker**	**Relevance**	**Technique**	**Sample**
Guo et al. (48)	-Poly (C)-binding protein 1 (PCBP1)	Oxaliplatin resistance	2D gel electrophoresis followed by MALDI TOF/TOF mass spectrometry	Cell lines and tumoural tissue
Martin et al. (49)	- Apolipoprotein E 180 (APOE) - Angiotensinogen (AGT) - Vitamin D binding protein (DBP)	Survival outcomes in patients treated with bevacizumab	Gel electrophoresis (2D-DIGE), followed by LC-MS/MS	Serum
Katsila et al. (50)	- Phosphorylated EGFR (pEGFR)	Response to Cetuximab	Quantitative proteomic analysis	Plasma
Yang et al. (60)	- Proteasome subunit alpha type 1 (PSA1 - Leucine aminopeptidase 3 (LAP3) - Annexin A3 (ANXA3) - Maspin (serpin B5)	Proteomic profiling of antibody-inducing cancer-associated immunogens	Mass spectrometry to evaluated antibody-reactive proteins. Western blotting and immunohistochemistry validation	Serum and CRC tissue
Chauvin et al. (64)	- Interferon induced protein with tetratricopeptide repeats 1 (IFIT1) - FAST Kinase Domains 2 (FASTKD2) - Phosphatidylinositol-5-phosphate 4-kinase type-2 beta (PIP4K2B) - AT-rich interactive domain-containing protein 1B (ARID1B) - Solute carrier family 25 member 33 (SLC25A33) - Caldesmon 1 (CALD1) - Carboxypeptidase A3 (CPA3) - Beta-1,3-galactosyltransferase 5 (B3GALT5) - CD177 - Receptor-interacting serine/threonine-protein kinase 1 (RIPK1)	Response to neoadjuvant chemoradiotherapy in rectal cancer	Mass spectrometry	FFPE CRC tissue

## Prognostic Biomarkers

A prognostic biomarker can be defined as a biological characteristic that gives information about the patient's overall cancer outcome, independent of therapy (65). The current staging strategy for colorectal cancer is the TNM system, which consists of the analysis of tumor depth of invasion (T), nodal involvement (N), and presence of metastasis (M) (66). The overall prognosis is determined by a combination of clinical and pathologic variables; however, the prognosis can be different between patients in the same stage, and, in some cases, patients at early stages can present poorer outcomes than patients at advanced stages. These variations are the result of a complex process of colorectal carcinoma (CRC) pathogenesis that involves multistep molecular pathways, initiated by genetic and epigenetic events (19).

The main prognostic biomarker used in clinical practice is carcinoembryonic antigen (CEA), a high-molecular-weight glycoprotein expressed in embryonic tissue and colorectal malignancies. This antigen was discovered in 1965, but it remains the most widely used blood-based biomarker for CRC. Elevated levels are associated with cancer progression and can indicate recurrence after surgery. However, high CEA levels are not specific to CRC and can also be found in other malignancies and inflammatory conditions, such as inflammatory bowel disease, liver disease, and pancreatitis (67, 68).

Recently, other parameters have been used to determine the prognosis. The effect of microsatellite instability (MSI) and BRAF mutation on survival in colorectal carcinoma was elucidated, and these genetic markers already have clinical applications (19). Despite these recent advances, additional prognostic biomarkers are urgently needed to optimize the management and follow-up of colorectal cancer patients.

The presence of metastases represents the main unfavorable prognostic factor in patients with colorectal cancer. The estimated 5-year survival for stage IV patients is ~8% (69). The major site of metastases in colorectal cancer is the liver, occurring in 20–35% of patients at the time of diagnosis and in nearly 70% of patients during the course of the disease (70). Marfà et al. (71) used a high-throughput proteomic technique to predict 5-year survival in patients with colorectal cancer who developed liver metastases. Human hepatic tumor samples were analyzed by surface-enhanced laser desorption/ionization time-of-flight mass spectrometry (SELDI), and a classification and regression tree analysis was done posteriorly. This approach allowed the identification of four relevant protein peaks and the construction of an algorithm that revealed an excellent diagnostic accuracy in differentiating mild from severe colorectal liver metastases patients (71).

Recently, Kirana et al. (72) performed a combination of laser microdissection, 2D-DIGE and MALDI-TOF MS to identify proteins associated with colorectal cancer spread. Initially, laser microdissection was applied to isolate cancer cells from primary colorectal tumors of stage II patients in two distinct groups: (i) patients who presented metastases within 5 years of initial surgical intervention and (ii) patients who did not present metastases within 5 years of initial surgical intervention. Then, 2D-DIGE (a technique that uses fluorescent dyes to label different conditions) and MALDI-TOF were used to identify the global profile of proteins, with posterior validation achieved through tissue microarray (TMA) immunohistochemistry. The expression of HLAB, 14-3-3β protein, a disintegrin and metalloproteinase with thrombospondin motifs (ADAMTS2), latent transforming growth factor beta binding protein 3 (LTBP3), nucleoside diphosphate kinase 2 (NME2), and jagged 2 protein (JAG2) was associated with clinical pathological parameters related to tumor progression, invasion, and metastasis (72).

Zhu et al. (73) used another approach, based on magnetic bead-based fractionation coupled with mass spectrometry, to compare serum samples from patients with metachronous liver metastases vs. patients without recurrence or metastases for at least 3 years after radical colorectal surgery. Serum proteomic fingerprinting was done, and it exhibited a promising value for predicting metachronous liver metastases in patients who underwent radical resection of colorectal cancer. The peptides were recognized as fragments of alpha-fetoprotein, complement C4-A, fibrinogen alpha, eukaryotic peptide chain release factor GTP-binding subunit ERF3B, and angiotensinogen (73).

The collagen proteins seem to be promising candidates as biomarkers in the metastatic scenario of colorectal cancer. A recent MS-based proteomic approach compared colorectal liver metastasis tissues with healthy adjacent liver tissues, demonstrating the upregulation of 19 of 22 collagen-α chains in colorectal liver metastasis tissue. Posterior validation with immunohistochemistry showed significant upregulation of collagen type XII in the metastatic context (74).

Some studies have also demonstrated the possibility of detecting colorectal liver metastases through the identification of collagen peptides in urine (75, 76). Urine is an interesting potential source of biomarkers as this biological fluid is easy to obtain non-invasively (24). An example of a promising application of this method is the measurement of the urinary prostaglandin metabolite PGE-M. PGE-M is the major urinary metabolite of prostaglandin E2, which plays an important role in mediating the effects of cyclooxygenase-2 in colorectal carcinogenesis. Elevated urinary levels of PGE-M seem to be correlated with advanced adenomas and an elevated risk of colorectal cancer (77–81).

The determination of nodal status in CRC is another point that requires new candidate prognostic biomarkers. Lymph node involvement results in poor prognosis, reducing the 5-year survival rate from 70 to 80% in patients with node-negative disease to 30%−60% in those with node-positive disease (82). The current non-invasive imaging methods used for the preoperative detection of lymph node metastasis, such as computed tomography, magnetic resonance imaging, and endorectal ultrasound, have low accuracy rates (83).

Non-invasive methods to predict the nodal status could improve the management of patients with colorectal cancer, guiding the indication of chemotherapy or the extension of the surgery. Mori et al. (84), in a recent study, used isobaric tags for relative and absolute quantitation (iTRAQ) as part of a proteomic analysis that identified 60 differentially expressed proteins specifically related to lymph node metastasis in patients with colorectal cancer. The validation process by immunohistochemistry revealed that heat shock protein 47 (HSP47) expression in colorectal cancer tissue was significantly higher than in adjacent normal colonic mucosa (84).

In another study by the same group, iTRAQ was used in a comparative proteomics approach, demonstrating that a high level of ezrin protein was an independent predictor of lymph node metastasis in colorectal cancer (85). The ezrin protein seems to occupy an important place in the carcinogenesis process, being described as a biomarker candidate for the progression and prognosis of gastrointestinal cancers and a target for anti-metastatic therapy (86, 87). Furthermore, some studies associate the upregulation of ezrin expression with rectal cancer recurrence and tumor aggressiveness (88, 89).

One of the most concerning points in the postoperative follow-up of CRC is recurrence detection. After surgery with curative intent, 30–40% of the patients present locoregional recurrence or distant metastasis (90). Regarding the discovery of prognostic biomarkers of colorectal cancer recurrence, Clarke et al. (91), in a recent study, used a reverse phase protein array to unveil the functional proteome in 263 colorectal cancer tumor samples from patients treated at MD Anderson Cancer Center and 462 primary tumor tissue from The Cancer Genome Atlas archived colorectal tumor bank. On multivariate analysis, eight proteins demonstrated significant prognostic factors for tumor recurrence: collagen VI, forkhead box O3, inositol polyphosphate-4-phosphatase, LcK tyrosine kinase, phospho-PEA15 (Ser116), phospho-PRAS40, Rad51, and phospho-S6 (Ser240-244) (91).

The expression of maspin was also described as a marker for early recurrence in stage IV colorectal cancer. Snoeren et al. (92) analyzed tumor tissue samples from five stage IV patients with early recurrence (<6 months) and five patients with prolonged time to recurrence (>24 months) through mass spectrometry with subsequent validation by Western blotting. They demonstrated that maspin was differentially expressed in stage IV colorectal cancer patients with early and late recurrence after surgery for colorectal liver metastases (92).

Despite the cited potential prognostic biomarkers (Table 3), carcinoembryonic antigen remains the only established protein biomarker in clinical practice to determine prognosis. The identification, validation, and translation of new prognostic biomarkers is important to fill the presented gaps in our knowledge, such as the prediction of nodal status, distance metastasis, and postsurgical recurrence.

**Table 3:** Examples of candidate prognostic biomarkers.

**References**	**Biomarkers**	**Technique**	**Sample**
Kirana et al. (72)	- HLAB - 14-3-3β protein - Disintegrin and metalloproteinase with thrombospondin motifs (ADAMTS2) - Latent transforming growth factor beta binding protein 3 (LTBP3) - Nucleoside diphosphate kinase 2 (NME2) - Jagged 2 protein (JAG2)	Combination of laser microdissection, 2D-DIGE and MALDI-TOF MS, with posterior validation through tissue micro array (TMA) immunohistochemistry.	CRC tissue
Zhu et al. (73)	- Fragments of alpha-fetoprotein - Complement C4-A - Fibrinogen alpha - Eukaryotic peptide chain release factor GTP-binding subunit ERF3B - Angiotensinogen	Magnetic bead-based fractionation coupled with MS.	Serum
Van Huizen et al. (74)	Collagen type XII	Mass-spectrometry	Colorectal liver metastasis tissues
Mori et al. (84)	Heat shock protein 47 (HSP47)	iTRAQ with validation by immunohistochemistry	Tumor tissue
Mori et al. (85)	Ezrin protein	iTRAQ	Tumor tissue
Clarke et al. (91)	- Collagen VI - Forkhead box O3 - Inositol polyphosphate-4-phosphatase - LcK tyrosine kinase, phospho-PEA15 (Ser116) - Phospho-PRAS40 - Rad51 - Phospho-S6 (Ser240-244)	Reverse phase protein lysate microarray(RPMA)	Tumor tissue
Snoeren et al. (92)	Maspin	MS, with subsequent validation by Western blotting	Tumor tissue

## Conclusion

The approach to patients with colorectal cancer has been dramatically updated recently thanks to a better understanding of the process of carcinogenesis and advances in the field of genetics. The determination of KRAS, BRAF, and MSI status has become an indispensable step in therapeutic planning, especially in patients with metastatic disease. Furthermore, the emergence of immunotherapy and the increasing use of liquid biopsies have extended the possibilities in the decision-making process toward personalized medicine. However, even with these advances, there is a lack of biomarkers that can guide the early diagnosis or the targeted treatment, prognosis, and surveillance of patients with colorectal cancer. Despite the improvements in MS technologies and the large number of proteomics-based studies to find biomarkers, this approach is not mainstream, especially regarding the management of colorectal cancer patients. This difficulty in translating protein markers into clinical practice is probably due to the small sample sizes of studies and to the heterogeneity in the processes of sample obtainment, preparation, and storage. These factors, coupled with complexities of data analysis and interpretation of proteomic approaches, result in poor reproducibility of the studies. However, the greatest limitation is related to the absence of validation of the possible biomarkers in large cohorts, comparing the data with the current methods of diagnosis, prediction, and prognosis. In this scenario, translational proteomics remains a powerful and promising tool for the discovery of biomarkers that can lead to important changes in the management of patients with colorectal cancer. Probably, the key to personalized medicine in colorectal cancer relies on studies that can integrate genomic, transcriptomic, and proteomic data, from a multiomics point of view, in the search for a biomarker panel that combines strong clinical data and accurate molecular findings.

## Author Contributions

BA has contributed to the drafting, writing, conceptualizing, and final revision of the manuscript. GB and IC have contributed to the writing, organizing of data and tables, and reference revision. MM has contributed to early drafting, writing, and reference surveys. PO has contributed to the conceptualizing of the manuscript. AM has contributed to the conceptualizing, writing, final revision, and supervision of the manuscript. All authors approved the review final form.

### Conflict of Interest

The authors declare that the research was conducted in the absence of any commercial or financial relationships that could be construed as a potential conflict of interest.
